# BipoJet Scissors as an Alternative to Laser in Lung Wedge Resection

**DOI:** 10.7759/cureus.60088

**Published:** 2024-05-11

**Authors:** Georgi Prisadov, Katrin Welcker, Kerstin Herrmann, Emeka B Kesieme, Albert Linder

**Affiliations:** 1 Department of Thoracic Surgery, Academic Teaching Hospital Maria Hilf, Mönchengladbach, DEU; 2 Institute of Pathology, Academic Teaching Hospital Bremen, Bremen, DEU; 3 Department of Cardiothoracic Surgery, Irrua Specialist Teaching Hospital, Irrua, NGA; 4 Department of Cardiothoracic Surgery, Castle Hill Hospital, Cottingham, GBR; 5 Department of Thoracic Surgery, Hospital St. Anna, Luzern, CHE

**Keywords:** surgical equipment, porcine lung, laser, bipojet scissors, lung wedge resection

## Abstract

Introduction: An important goal in every lung resection is airtight closure of the resected lung surface. This can be achieved with several techniques, including clamp resection, stapler, laser, and various high-frequency methods. By quantitatively measuring the air fistula across the resection surface of porcine lungs, two resection techniques were compared in our study: BipoJet dissecting scissors (Aesculap, Inc., Tuttlingen, Germany) and laser (Ceralas HPD®, Biolitec Inc., Jena, Germany).

Methods: Following a stencil, wedge resections were performed in porcine lungs using water-irrigated bipolar scissors and laser (1350 nm, 40 watts, non-contact mode). The volume of the air fistula was then measured. The irrigation technique involved the attachment of an irrigation channel to a pair of standard surgical scissors. A sodium chloride (NaCl) solution was fed at a defined flow rate, along the blades of the scissors onto the parenchyma. This technique was used on a total of 10 specimens each.

Results: Somewhat better pneumostasis was achieved with laser resection, though the difference was small and not statistically significant. The flow rate was 124 mL/min/cm² after laser resection and 145 mL/min/cm² after using the BipoJet scissors. The difference was not statistically significant. Water irrigation during resection with the BipoJet scissors prevents the temperature in the tissue from exceeding 100°C thus avoiding tissue carbonization. These scissors offer the following advantages: ease of use, no need to change instruments, no need for staff training, no protective measures, all-in-one incision/coagulation/dissection, low cost, and a clear surgical field due to the irrigation effect.

Conclusions: Resection of lung parenchyma, e.g., during resection of metastases, is easier with BipoJet scissors and comparable to laser resection. This was established both experimentally and by resecting lung metastases.

## Introduction

At present, a typical resection of lung parenchyma (wedge resection, enucleation) generally involves the use of clamps or staplers, various high-frequency methods, laser, or ultrasound. Airtight closure of resected lung surfaces after wedge or segmental resections is recognized to have a considerable influence on postoperative recovery.

No scientific data or generally valid guidelines are available concerning the method for sealing the metastatic area (suturing or sealing with thermal, light, or ultrasound energy). Only clinical studies into fibrin glue or other tissue adhesives have been performed [1-3].

The technique of metastasectomy is very variable and depends more upon the surgical school, surgical training, and personal preferences than upon the available evidence [4].

At present, Nd:YAG (neodymium-doped yttrium aluminum garnet) lasers are increasingly used to resect lung metastases. Laser metastasectomy significantly reduces parenchymal loss, but its effect on long-term survival remains unclear [5-7].

Lung parenchyma resection involves various high-frequency methods, laser, or ultrasound, whereby electrical energy, light, or ultrasound waves are converted into thermal energy. The cells thus heat up suddenly, causing them to rupture and tear apart the cell aggregate. If the tissue is heated with the applicator to temperatures above 180ºC, the tissue proteins carbonize [8,9]. It is preferable to avoid this situation given that it creates an excessively high rate of air leakage and presumably delays the spontaneous healing of the resected surface [10]. Based on our clinical experience, we use bipolar scissors to resect metastases. To control the tissue temperature, the cutting blades and the tissue are continuously irrigated with water.

In our experimental study, we used an ex vivo porcine model to compare air loss after lung parenchyma resection using BipoJet scissors (Aesculap, Inc., Tuttlingen, Germany) and laser.

## Materials and methods

Study design, materials, and ventilation

The study involved a scaled-down design as described for the isolated human lung perfusion (IHLP) model [11]. However, the perfusion model was not required for our research purposes.

**Figure 1:**
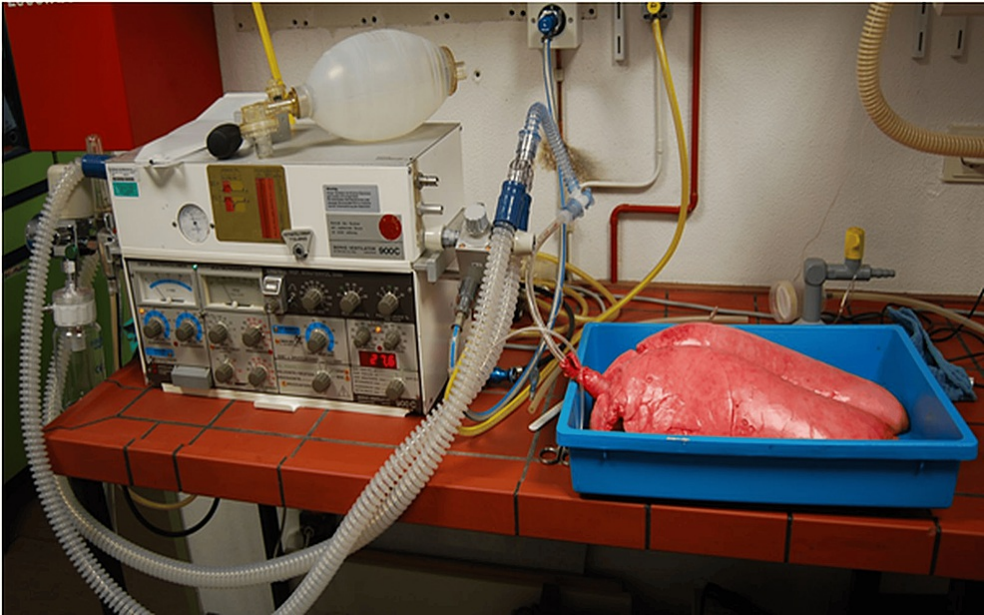
Test construction: intubated and connected via the trachea to ventilate the lungs.

Lungs of pigs slaughtered within four hours were fastened in pairs at the trachea, then aspirated by means of bronchoscopy, intubated, and connected via the trachea to a ventilator (Siemens-Elema AB SV 900C, Munich, Germany) (Figure 1).

With the ventilatory parameters set at a flow rate of 10 L/min and a frequency of 20/min, the respiratory pressures remained within the physiologic range at pmax = 20 mbar. The specimens did not appear to be over-distended.

Protocol

In a double-armed study, wedge resections with a diameter of 2 cm were created using a stencil. The air fistula volumes over the defects were then measured under defined ventilatory parameters.

**Figure 2:**
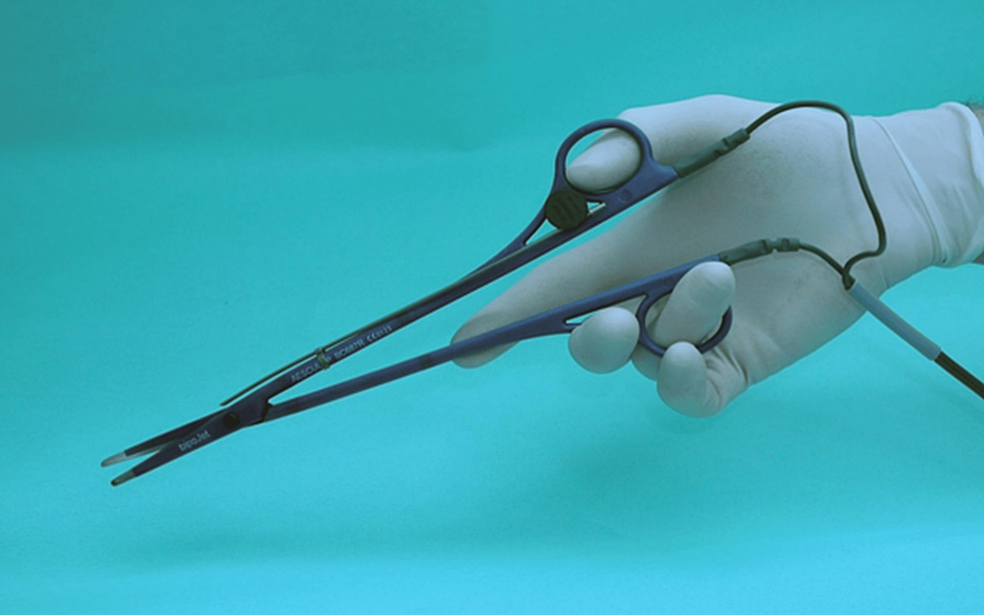
BipoJet scissors with an irrigation cannula attached to the blades of the scissors.

In the first series of measurements, the wedge resections were carried out with a 1350 nm laser light of 40 watts in continuous non-contact mode (Ceralas HPD®, Biolitec AG, Jena, Germany). In the second series, the wedge resections were performed using standard surgical bipolar scissors with water irrigation (Aesculap AG, Tuttlingen, Germany). The scissors operate under a modified high-frequency current. With a channel attached to the blades of the scissors, the water flowed onto the resected tissue surface at a rate of 1.5 mL/min (Figure 2).

The Erbe VIO high-frequency generator (Erbe Inc., Tübingen, Germany) was set to a maximum output of 60 watts, with an effect level of 5, in “Bipo-Soft” mode.

Air fistula measurement

**Figure 3:**
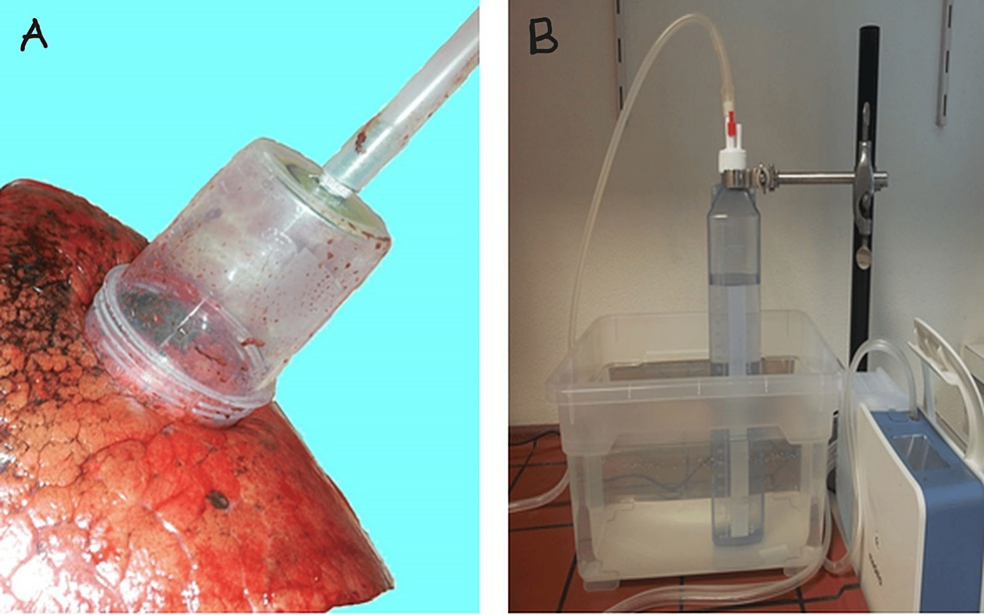
The air escaping through the defect is channeled through a funnel and collected in a water-filled displacement receptacle. A: Funnel over lung defect. B: Water-filled displacement receptacle.

The air escaping the defect was channeled through a funnel and collected in a water-filled displacement receptacle (Figure 3).

The amount of water displaced/time corresponds to the amount of air escaping through the air fistula volume. Measurement lasted 10 seconds and the shape of the container ensured that the pressure required to achieve displacement would not distort the free fistula volume. This simple configuration enables accurate measurement of the fistula volume with a time delay of only a few seconds.

Statistical analysis

In all five pairs of lungs, four wedges were resected from the upper and lower lobes. Each of these five defective pairs was assigned to a series. The average values per cm² and the standard deviations of the air fistulas from both series were compared.

The significance of the fistula volume in both series was analyzed statistically. The purpose of the statistical analysis was to compare both groups for average fistula volume. A t-test or analysis of variance (ANOVA) could be used but the necessary conditions (normal distribution, homogeneity of variance, and sample size) were not fulfilled in this case. We therefore employed a modified method that accounts for the deviation in the distributional form of the observations. The Monte Carlo method was used to generate a sufficiently large sample size with the distributional form as in the original samples [12]. This facilitates the construction of test statistics that correspond to the real distribution. This distribution was used to assess the p-value, i.e., the critical value relevant to test interpretation.

## Results

**Figure 4:**
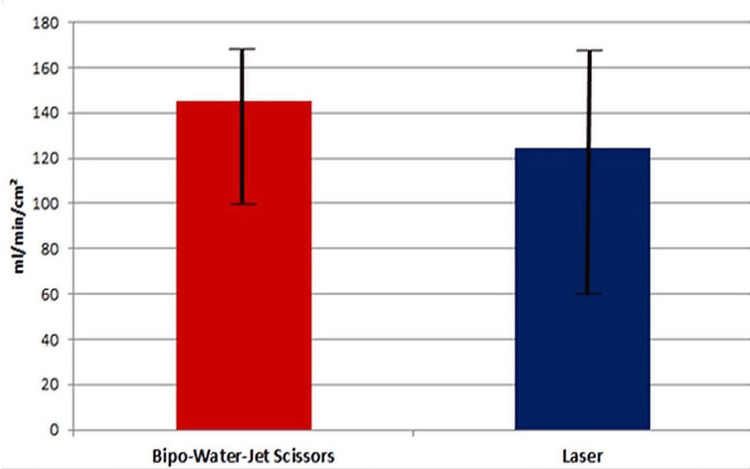
Statistical analysis: air fistula rate (ml/min/cm²)

The average air fistula volumes were 124 mL/min/cm² in series 1 (laser resection) and 145 mL/min/cm² in series 2 (resection with BipoJet scissors) (Figure 4).

There was a slight difference between the two series in favor of laser resection. Based on the statistical analysis, however, the average values for the air fistulas were not found to differ significantly in either series (α = 5%, Welch’s ANOVA, p-value = 0.63).

**Figure 5:**
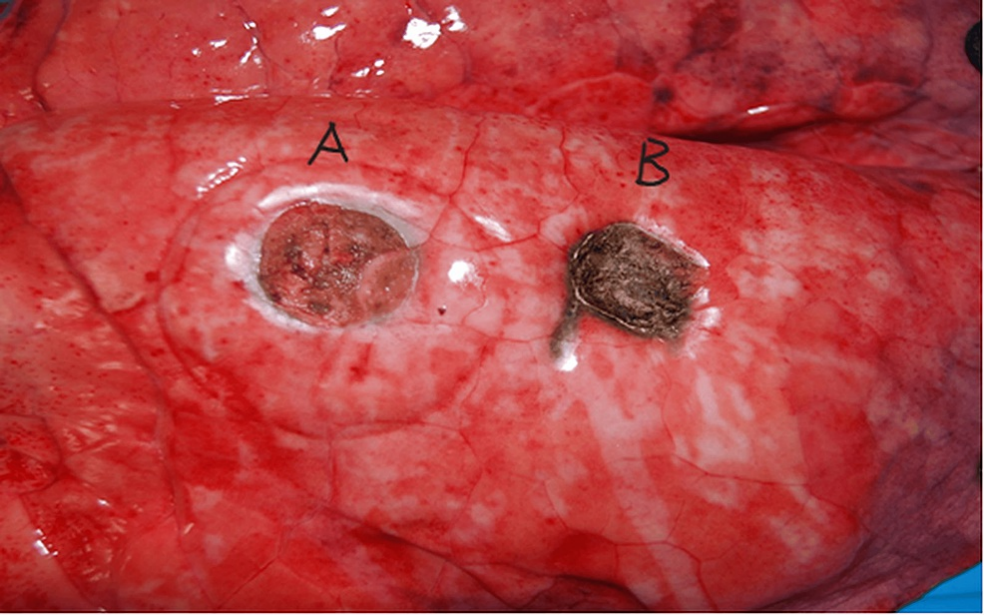
Macroscopic view of the resected lung surface A: Bipo-Water Jet scissors. B: Laser.

During the wedge resection with the BipoJet scissors, a smooth, reflective barrier appeared on the surface of the parenchymal defect, which resembled neopleura rather than the carbonization on the resected surface that is typical of laser resections (Figure 5).

The experiments were designed in such a way that no inter-individual variations arose between the specimens when comparing the measured values of both series. The two methods were always compared separately; the wedge resections in the upper and lower lobes, respectively, were compared with each other.

Histology

**Figure 6:**
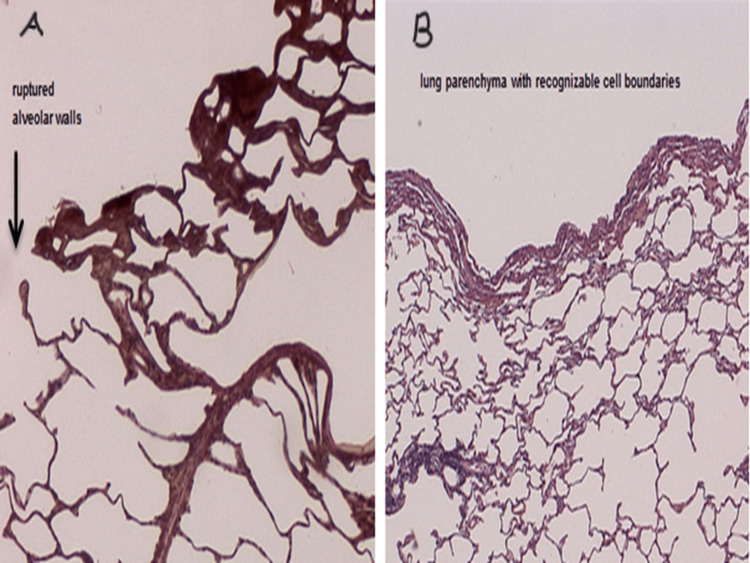
Histological examination performed on hematoxylin and eosin sections from the surface of the resected portions of the lung A: After laser resection. B: After resection with Bipo-Water-Jet scissors.

Histological examination was performed on hematoxylin and eosin sections from the surface of the resected portions of the lung. Frequently ruptured alveoli and some carbonization zones were found in the specimens of series 1 (laser); mainly coagulation zones and only a few ruptured alveolar walls were found in series 2 (bipolar water-jet scissors) (Figure 6).

It should be noted that during the processing of the resected material, coal aggregates were washed out. Hence, in thin sections, significantly fewer carbon particles can be seen with the naked eye after laser incision.

## Discussion

The visceral pleura is opened in every lung resection, potentially creating the ideal conditions for air to escape from the lungs into the pleural space. Clinical experience has shown that the severity of the fistulas in the uncovered parenchyma depends upon the resection technique. Sharp dissection of the tissue with a scalpel or scissors leads to severe fistulization whereas tissue resection by means of high-frequency cauterization or laser results in less severe fistulization because heat is applied simultaneously.

It is also clear from clinical experience with high-frequency surgery that the thermal effect of this resection technique promotes hemostasis while also reducing the extent of the air fistulas.

We have previously reported that overheating of tissue (>180°C) leads to carbonization. The pneumostatic effect is thus decreased and air fistulas increase in size [10].

It is therefore desirable to control the tissue temperature. This was achieved in this experiment by irrigating the blades of the scissors and the lung tissue simultaneously. The plateau of the temperature curve for water at 100°C due to evaporation prevented any further rise in temperature. Proteins are denatured at this temperature, i.e., coagulation is observed at the macroscopic level. This will evidently lead to relative sealing of the resected surface and consequently both hemostasis and pneumostasis.

Under ideal conditions, a smooth reflective barrier forms on the surface of the parenchymal defect following wedge resection with the BipoJet scissors. Resembling neopleura, no carbonization is noted on the resected surface as is typical of resection with a laser in non-contact mode. Irrigation of the cutting blades with water during resection also has the advantage that blood is removed from the resection site. This allows for a consistently good view of the tissue to be resected as well as the tumor margins. Another advantage of irrigating with water is that undesirable adhesion of lung tissue and blood to the cutting blades and crusting at the tips of the scissors are prevented.

Laser resection of lung metastases has long been promoted in the scientific literature [7,13] as well as in the lay press and on the internet [14,15]. Laser metastasectomy significantly reduces the loss of parenchyma, although the oncologic benefit of laser resection over high-frequency resection or conventional clamp resection has never been proven to be significantly superior [5,6,10].

The literature states that the increase in temperature occurring in the surrounding lung tissue during laser resection allegedly adds an additional oncologic gain in terms of safety margins [10].

Osei-Agyemang et al. reported a significantly higher rate of pneumonia from thermally induced pneumonitis because the laser causes heat to develop in the dissection plane during multiple resections of metastases [6]. We believe that the risk of thermal pneumonitis in the case of multiple metastatic resections is minimal when the tissue is cooled with water.

We found no similar study that compared the use of BipoJet scissors to laser in lung resection but there were studies comparing LigaSure (bipolar electrosurgical instrument) versus conventional electrocautery, monopolar cutter versus laser, and stapler versus laser technique in pulmonary resection [16-18]. The use of a monopolar cutter was associated with more adjacent tissue injury than the laser technique, which could invariably result in an increased risk of fistula and air leak [17]. The bipolar device was also superior to conventional electrocautery [16].

In our experience, the BipoJet scissors demonstrated several advantages during the resection of multiple metastases. They include ease of use (no new instruments), no need to change instruments, all-in-one incision/coagulation/dissection, no need for personnel training, no protective measures, and low cost. This method can also be used for (tumor) decortication.

The limitation of the study is that the experiment was performed on porcine lungs within four hours of demise.

## Conclusions

Metastatic resection with BipoJet scissors is a technique that is simple and cost-effective but of equal value to laser resection in terms of pneumostasis as the difference in flow rates between the two techniques has been found not to be statistically significant. In addition, irrigating with water prevents the carbonization of tissue and will present a clear surgical field.

With the BipoJet scissors, the surgeon can prepare, cut, and coagulate at the same time. This has been demonstrated not only experimentally but also during daily clinical resection of lung metastases.
